# Essential Gene Clusters Involved in Copper Tolerance Identified in *Acinetobacter baumannii* Clinical and Environmental Isolates

**DOI:** 10.3390/pathogens9010060

**Published:** 2020-01-15

**Authors:** Rapee Thummeepak, Renuka Pooalai, Christian Harrison, Lucy Gannon, Aunchalee Thanwisai, Narisara Chantratita, Andrew D. Millard, Sutthirat Sitthisak

**Affiliations:** 1 Department of Microbiology and Parasitology, Faculty of Medical Science, Naresuan University, Phitsanulok 65000, Thailand; rapee_worm32@hotmail.com (R.T.); tingtang34166@hotmail.com (R.P.); Aunchaleet@nu.ac.th (A.T.); 2 Department of Genetics and Genome Biology, University of Leicester, University Road, Leicester LE1 7RH, UK; cdh18@leicester.ac.uk (C.H.); lg248@leicester.ac.uk (L.G.); adm39@leicester.ac.uk (A.D.M.); 3 Department of Microbiology and Immunology, Faculty of Tropical Medicine, Mahidol University, Bangkok 10400, Thailand; narisara@tropmedres.ac; 4 Mahidol-Oxford Tropical Medicine Research Unit, Faculty of Tropical Medicine, Mahidol University, Bangkok 10400, Thailand

**Keywords:** *Acinetobacter baumannii*, copper susceptibility, copper-related genes, whole-genome sequence analysis, gene expression

## Abstract

Copper is widely used as antimicrobial in agriculture and medicine. Copper tolerance mechanisms of pathogenic bacteria have been proven to be required for both copper tolerance and survival during bacterial infections. Here, we determined both copper-tolerant phenotype and genotype in *A. baumannii* originated from clinical and environmental samples. Using copper susceptibility testing, copper-tolerant *A. baumannii* could be found in both clinical and environmental isolates. Genotypic study revealed that representative copper-related genes of the cluster A (*cueR*), B (*pcoAB*), and D (*oprC*) were detected in all isolates, while *copRS* of cluster C was detected in only copper-tolerant *A. baumannii* isolates. Moreover, we found that copper-tolerant phenotype was associated with amikacin resistance, while the presence of *copRS* was statistically associated with *bla*_NDM-1_. We chose the *A. baumannii* strain AB003 as a representative of copper-tolerant isolate to characterize the effect of copper treatment on external morphology as well as on genes responsible for copper tolerance. The morphological features and survival of *A. baumannii* AB003 were affected by its exposure to copper, while whole-genome sequencing and analysis showed that it carried fourteen copper-related genes located on four clusters, and cluster C of AB003 was found to be embedded on genomic island G08. Transcriptional analysis of fourteen copper-related genes identified in AB003 revealed that copper treatment induced the expressions of genes of clusters A, B, and D at the micromolar level, while genes of cluster C were over-expressed at the millimolar levels of copper. This study showed that both clinical and environmental *A. baumannii* isolates have the ability to tolerate copper and carried numerous copper tolerance determinants including intrinsic copper tolerance (clusters A, B, and D) and acquired copper tolerance (cluster C) that could respond to copper toxicity. Our evidence suggests that we need to reconsider the use of copper in hospitals and other medical environments to prevent the selection and spread of copper-tolerant organisms.

## 1. Introduction

Copper is an essential trace metal that is required for many biological processes such as electron transports, structural stabilizations of proteins, and oxidative respirations in bacteria [1]. Although copper is important for bacterial growth, an excessive amount is toxic to the cells, leading to the production of reactive oxygen species (ROS), resulting in oxidative damage to biomolecules [2]. The toxic effect of copper on microbial cells is utilized in agriculture and medicine as an antimicrobial agent that could lead to the emergence of copper-tolerant strains [1,2]. Bacteria can protect themselves from the toxic effects of copper through different mechanisms such as cellular sequestrations, active efflux systems, and detoxifications by copper oxidase enzymes [1,3,4]. Moreover, copper tolerance genes have been reported in many bacteria and are involved in virulence and pathogenesis [5]. For example, during bacterial infections, phagocyte increases the expression of copper transporter genes that transfer copper into a phagolysosome to kill the bacterial cells [5]. Recent studies revealed that copper ions are mobilized to urine during a urinary tract infection to control *E. coli*, while bacterial cells elevate the expression of copper tolerance genes to protect themselves against toxic copper [6,7].

*Acinetobacter baumannii* is one of the major pathogenic bacteria that causes a wide range of infectious diseases in humans, which include wound infections, bacteremia, pneumonia, meningitis, and urinary tract infections [8,9]. *A. baumannii* normally inhabits water and soil, and has also been isolated from hospital environments [9,10]. This bacterium is capable of growing in a wide range of diverse environmental conditions and the genes involved in copper tolerance have been identified and characterized in clinical isolates [11,12,13,14]. Williams et al. [11] observed that *A. baumannii* harbored many putative copper-related genes located on chromosomal regions, while a comparative genome study revealed the region carrying *cop* genes of *A. baumannii* were found to be embedded on genomic islands (GEIs), namely G08 and G62 [15]. Previous studies reported that exposure of *A. baumannii* cells to copper caused overexpressions of the genes encoding the putative copper transporters (*actP1*, *actP2*, and *copB*), the putative copper regulator (*copR*) and the putative copper-binding proteins (*copD* and ABUW_2708) [11,12]. Recently, a functional analysis by Alquethamy et al. [14] showed that the copper efflux P-type ATPases mediates copper tolerance, and interestingly, this efflux pump also contributes to *A. baumannii* survival in a pneumonia murine model [14]. These supported that copper tolerance is essential for both cellular responses to copper toxicity and virulence of *A. baumannii*, whereas, the mechanism of copper tolerance in *A. baumannii* has only been studied in a limited number of isolates [11,12,13]. This study aims to examine phenotypic and genotypic tolerance to copper in a large collection of *A. baumannii* from both clinical and environmental origins, and to investigate the genotypic response to copper tolerance in a representative clinical *A. baumannii* isolate.

## 2. Materials and methods

### 2.1. Bacterial Strains and Growth Condition

A total of 394 clinical *A. baumannii* isolates used in this study were collected from different clinical samples obtained from 6 hospitals located in various geographical regions of Thailand. Natural environmental isolates of *A. baumannii* (13 isolates) were provided by Leungtongkam et al. [16]. Biochemical characterization and species confirmation were performed as previously described [16]. *A. baumannii* and *E. coli* (ATCC 25922) were grown in standard Mueller-Hinton or Luria Bertani (LB) media (HiMedia) at 37 ℃ with shaking (Table S1).

### 2.2. Antibiotic Susceptibility Testing and PCR Detection of Cabarpenemase Encoding Genes

To determine the antimicrobial susceptibility of *A. baumannii* isolates, the disc diffusion method was performed and interpreted according to the Clinical and Laboratory Standards Institute recommendations [17]. The following antibiotics were used in this study: amikacin (30 μg/disc), cefotaxime (30 μg/disc), ceftazidime (30 μg/disc), ceftriaxone (30 μg/disc), cefepime (30 μg/disc), ciprofloxacin (5 μg/disc), gentamicin (10 μg/disc), imipenem (10 μg/disc), meropenem (10 μg/disc), trimethoprim/sulfamethoxazole (1.25/23.75 μg/disc), tetracycline (30 μg/disc), cefoperazone/sulbactam (105 μg/disc), piperacillin/tazobactam (100/10 μg/disc), and tigecycline (15 μg/disc) (Oxoid, Basingstoke, UK). The presence of beta-lactamase encoding genes including *bla*_OXA-23_, *bla*_OXA-24_, *bla*_OXA-58_, and *bla*_NDM-1_ were detected using singlet PCR assay in all isolates of *A. baumannii* as described previously [16].

### 2.3. Minimum Inhibitory Concentration (MIC) of Copper

Susceptibility to copper was determined using the agar dilution method as described in the CLSI guideline [17]. The Mueller-Hinton agar plates were prepared with concentrations of copper sulphate (CuSO_4_·5H_2_O) (Merck, Darmstadt, Germany) of 0, 0.3125, 0.625, 1.25, 2.5, 5, 10, 20, 40, and 80 mM and adjusted to pH 7.0, using 1 M sodium hydroxide. Bacterial inoculum was prepared by growing each isolate in Mueller-Hinton broth until exponential growth was reached (4–5 h) and the cell density was adjusted to ∼10^7^ cells/mL. The plates were spot-inoculated with 1 μl of adjusted cell suspension of each bacterium to obtain bacteria at 10^4^–10^5^ cells/mL. The inoculated plates were incubated for 24 h at 37 ℃ to determine the MIC value, defined as the lowest concentration of copper that inhibits bacterial growth [17]. All MIC experiments were performed in duplicate and *E. coli* strain ATCC 25922 was used as a negative control.

### 2.4. Detections Of Copper-Related Genes in Collections of A. baumannii

In our collection, we searched for copper tolerance genes such as *cueR*, *pcoAB*, and *copRS* previously identified in *A. baumannii* strain AB5075_UW [11,14] (Table S2). In addition to previously identified copper tolerance genes, a gene encoded for copper utilization, OprC was also searched in this study (Table S2). We divided these putative copper-related genes (proposed genes conferring copper tolerance and utilization) of *A. baumannii* into four clusters on the basis of their orientation and location (Table S2). The genes encoding for CueR, PcoAB, CopRS, and OprC were selected as representatives of cluster A, B, C, and D, respectively. The specific primers were designed by using online software, Primer-BLAST (https://www.ncbi.nlm.nih.gov/tools/primer-blast/) (Table S3). To detect copper-related genes, PCR was performed with crude boiled-cell lysates prepared from both clinical and environmental *A. baumannii* isolates, as described previously [16].

*In silico* detection of representative copper-related genes was conducted to study the prevalence of these genes in NCBI genome database. Complete chromosome (153 chromosomes) and complete plasmid (273 plasmids) sequences of *A. baumannii* were retrieved from NCBI Genome database via www.ncbi.nlm.nih.gov/genome/genomes/403 (last accessed June 2019). Copper-related genes of *A. baumannii* strain AYE were used as nucleotide query sequences; *cueR* (ABAYE2550), *pcoAB* (ABAYE3110 to 3111), *copRS* (ABAYE3203 to 3204) and *oprC* (ABAYE3703). BLASTn was conducted with an e-value cutoff 1e-10, minimum 80% identity and 95% query coverage.

### 2.5. Exposure of A. baumannii Cells to Heavy Metals and Imipenem, and Transmission Electron Microscopy (TEM) Analysis

The representative *A. baumannii* strain AB0003 was used to evaluate the effect of copper exposure on cell morphology and expression of copper tolerance genes. This strain was used as representative based on its ability to tolerate copper and since it carried multiple copper-related clusters. This representative isolate was cultured to exponential phase as described above and then 5 mL was aliquoted. To challenge bacterial cells, the exponential culture was then distributed into 15 mL of pH-adjusted Mueller-Hinton broth containing various concentrations (0, 0.0048, 0.625, 1.25, 5, and 10 mM) of copper sulphate and then incubated for an additional 30 min. After 30-min incubation, copper-treated cultures were aliquoted for viable cell counting, TEM analysis, and total RNA extraction. The bacterial enumeration of each copper treatment was conducted using serial 10-fold dilutions and plate count method. The negative staining and TEM image analysis of copper-treated cells was performed as described previously [18]. The cell morphology of copper-treated cells was visualized under transmission electron microscopy (HT 7700, Hitachi, Japan).

To compare with the presence of a 1/8xMIC of copper (1.25 mM), the *A. baumannii* strain AB003 was also exposed to other metals and imipenem to determine an inducible expression of *oprC*. The exponential culture of AB003 (5 ml) was added into 15 mL of pH-adjusted Mueller-Hinton broth containing a 1/8xMIC of other metals such as 1.25 mM of zinc sulphate (ZnSO_4_·7H_2_O) (Sigma-Aldrich, St. Louis, MO, USA), 1.5 µM of mercury chloride (HgCl_2_) (Merck, Darmstadt, Germany), or 8 µg/mL of imipenem monohydrate (Sigma-Aldrich, St. Louis, MO, USA). After 30-min incubation, the metal- and imipenem-treated cells were collected for total RNA extraction.

### 2.6. Total RNA Isolation, cDNA Synthesis, and Quantitative Real-Time PCR (qPCR)

The challenged and control cells of *A*. *baumannii* were collected and kept at −80 °C until RNA isolation. Total RNA extractions were performed using the Total RNA Extraction Mini Kit (RBC Bioscience, Taiwan) following the manufacturer’s procedure. The RNA concentration was quantified by using a NanoDrop 1000 Spectrophotometer (Thermo Scientific, Waltham, MA, USA). cDNA was synthesized with 5 µg of total RNA by using the Tetro cDNA Synthesis Kit (Bioline, London, UK) according to the manufacturer’s instructions. qPCR was done with the ABI Prism (Applied Biosystems, Foster City, CA, USA) using the SYBR green assay (Sensifast SYBR No-Rox Kit, Bioline, London, UK). All primer sequences used for the qPCR experiment are included in Table S4. The independence triplicates of each experiment were performed. Relative gene expression was calculated by the delta-delta Ct (2^−∆∆Ct^), using the *gyrB* gene as the internal reference.

### 2.7. Whole Genome Sequencing and Genome Assembly

The representative isolate, AB003 was cultured onto an LB agar plate and incubated overnight at 37 °C. Genomic DNA was extracted using a Real Genomics DNA Extraction Kit (RBC Biosciences, New Taipei City, Taiwan). Extracted DNA was quantified by using a Qubit^®^ DNA Assay Kit in Qubit^®^ 2.0 Fluorometer (Life Technologies, Carlsbad, CA, USA). The purified genomic DNA was used to construct libraries followed by sequencing with the Illumina HiSeq 2500-PE125 platform at the Novogene Bioinformatics Technology Co., Ltd. (Novogene, Beijing, China). Quality trimming of illumina’s paired-end reads was done with default parameters using Sickle v1.33 [19]. After quality-based trimming, de novo assembly was performed using SPAdes genome assembler v3.12 with default settings [20], from which only contigs larger than 500 bp were chosen for further analysis. The correction of assembly errors was carried out using Pilon v1.22 with default options [21]. This whole genome sequencing project was deposited at the European Nucleotide Archive (ENA) under the BioProject number PRJEB32181.

### 2.8. Bioinformatic Analysis

The genome was orientated against chromosome of *A. baumannii* A85 (accession no. NZ_CP021782) using Abacas v1.3.2 with default settings [22], prior to annotation with Prokka v1.12 using default parameters [23]. Copper-related genes presented in AB003 were identified using BLASTp. Copper tolerance proteins in BacMet database, copper tolerance proteins identified in AB5075_UW [11,14], and copper utilization system OprC of *P. aeruginosa* PA01 [24] were used as queries for BLASTp searching. The predicted functions of identified copper-related genes of AB003 were determined using BLASTp against the BacMet database (experimentally confirmed copper tolerance genes) [25]. The *A. baumanii pco* and *cop* genes were manually curated using BLASTp searches against Pco (accession no. Q47452-7) encoded from pRJ1004 and Cop (accession no. P12374-7 and Q02540-1) encoded from pT23D. The prediction of subcellular localization and transmembrane domains was determined by using CELLO v2.5 [26], and PRED-TMMB [27], respectively.

### 2.9. Construction of Chromosomal Circular Map and Genomic Island Comparisons

The prediction of genomic islands (GEIs) was conducted by running BLASTn. A set of nucleotide query of GEIs identified in *A. baumannii* AYE was used for BLASTn searching [15]. Sequences with coverage of more than 85%, e-values less than 1e–10, and identity of more than 90% were considered as positive GEIs.

The circular map was constructed using BLAST Ring Image Generator (BRIG) v0.95 [28] to compare AB003 chromosome (ordered contigs) against other reference strains of *A. baumannii*. A comparison of genomic islands harbored copper-related genes detected in chromosome or plasmid was visualized using Easyfig v2.2.3 [29].

### 2.10. Data, Statistical, and Cluster Analysis

All data and statistical analysis were performed by using Stata (Stata12.0, Corporation, College Station, TX, USA). The comparisons of frequencies between two groups and among multiple groups were analyzed by Fisher’s exact and Chi-square tests, respectively. In the case of continuous variable analysis, a parametric Student’s t test to compare two groups or a One-Way ANOVA to compare multiple groups was used for data with the normal distribution. The *p*-values of less than 0.05 were regarded as statistically significant. For cluster analysis, the percentages of antibiotic resistance and copper tolerance of *A. baumannii* among difference sources were used as input data to construct the dendrogram using DendroUPGMA (http://genomes.urv.es/UPGMA/) [30] with default parameters.

## 3. Results

### 3.1. Prevalence of Copper-Related Genes in A. baumannii

Overall, representative genes of clusters A (*cueR*), B (*pcoAB*), and D (*oprC*) were detected in all isolates of *A. baumannii*, while cluster C (*copRS*) was detected in 84 out of 407 isolates (20.64 %) (Table 1 and Table S1). Positive rates of *copRS* between clinical and environmental isolates were 19.8% (6/13) and 46.15 % (78/394) respectively. To support this finding, we performed in silico detection of these genes in a collection of complete chromosome-sequenced *A. baumannii* retrieved from the NCBI database. The result showed that *cueR*, *pcoAB* and *oprC* were detected in all analyzed strains (Table S5). In the case of *pcoAB*, all strains were found to be carrying the intact full-length gene, except for two strains (accession no; CP026761.1 and CP040056.1) that carried IS*Aba1*-disrupted variants. In contrast, *copRS* could be detected in 43 of the 151 isolates examined (Table S5). Moreover, among representative genes, we found that only *copRS* was carried on two *A. baumannii* plasmids (2/273 plasmids) (Table S6).

### 3.2. Copper Susceptibility Profiles of A. baumannii and Their Association with Copper-Related Genes

Overall, out of the 407 *A. baumannii* isolates 96 (23.59%) were found to be copper-tolerant strains (MIC equal to 10 mM) (Table 1 and Table S1). Among 394 clinical isolates, 90 (22.84%) isolates showed an ability to tolerate copper, while six of 13 environmental isolates (46.15%) exhibited a copper MIC equal to 10 mM (Table 1 and Table S1).

The relationship between genotypic and phenotypic tolerance to copper was analyzed by using Fisher’s exact test analysis (Table 1). Among all isolates of *A. baumannii*, two patterns of copper-related genes (*cueR*^+^/*pcoAB*^+^*/copRS*^-^*/oprC*^+^ and *cueR*^+^/*pcoAB*^+^*/copRS*^+^*/oprC*^+^) were detected and the presence of *copRS* was associated with copper-tolerant phenotype of *A. baumannii* (Table 1). Overall, the majority of copper-tolerant isolates were found to harbor *copRS* (84/96 isolates, 87.5%), while no copper-susceptible isolates were positive for *copRS* (0/311 isolates) (*p* < 0.001). In environmental isolates, all of the copper-tolerant isolates were positive for *copRS*. Likewise, in clinical isolates, ~ 86.7% of the copper-tolerant isolates (78/90 isolates) were positive for *copRS*, while all of the copper-susceptible isolates were negative for the detection of *copRS* (0/304 isolates) (*p* < 0.001).

### 3.3. Association between Copper Tolerance and Antibiotic Resistances in A. baumannii

**Figure 1:**
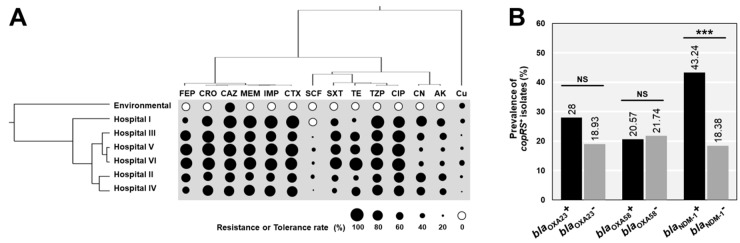
Relationship of antibiotic resistance and copper tolerance among *A. baumannii* strains. (**A**) The cluster analysis of drug and copper susceptibility profiles among *A. baumannii* obtained from different sources. (**B**) The statistical association of beta-lactamase genes and *copRS* in all tested isolates. AK: amikacin, CTX: cefotaxime, CAZ: ceftazidime, CRO: ceftriaxone, FEP: cefepime, CIP: ciprofloxacin, CN: gentamicin, IMP: imipenem, MEM: meropenem, SXT: trimethoprim/sulfamethoxazole, TE: tetracycline, SCF: cefoperazone/sulbactam, and TZP: piperacillin/tazobactam. *** *p*-value < 0.001 was considered as a significant difference (Fisher’s exact test). NS: not statistically significant.

A total of 407 non-duplicated *A. baumannii* isolates were tested for their antibiotic susceptibilities and their antibiogram compared with copper tolerance (Figure 1). The antibiotics-resistant and copper-tolerant profiles of *A. baumannii* derived from various sources were clustered to study their relationships (Figure 1A). Copper tolerance clustered outside the clade of antibiotics, supporting that copper tolerance was less associated with antibiotic resistance of *A. baumannii* (Figure 1A). Likewise, the percentages of copper-tolerant isolates between *A. baumannii* resistant and susceptible to each antibiotic showed no statistically significant difference (*p*-values > 0.05). Except for amikacin, 54 of 176 amikacin resistance strains (30.68%) were tolerant to copper, while only 42 of 231 (18.18%) of amikacin susceptible strains were found to be copper-tolerant strains (*p* < 0.01).

We also investigated the association between the presence of beta-lactamase genes and the acquired copper-related genes, *copRS*. There were no statistical differences of *copRS* positive rates detected in strains carrying *bla*_OXA-23_ or *bl_a_*_OXA-58_ compared with the *bla*_OXA-23_ negative or *bl_a_*_OXA-58_ negative strains (Figure 1B). However, the prevalence of *copRS* in *bla*_NDM-1_ positive strains was significantly higher than that in negative strains (43.24 vs. 18.38%; *p* < 0.001) (Figure 1B).

### 3.4. Visualization of A. baumannii AB003 Challenged with Copper

**Figure 2:**
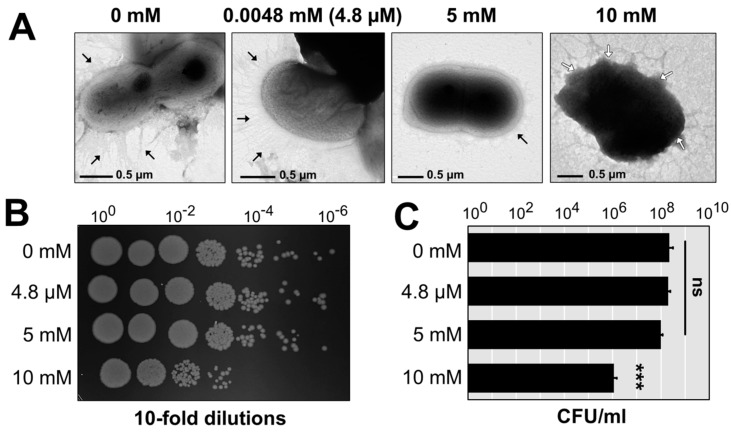
External morphology of *A. baumannii* strain AB003 treated with various concentrations of copper. (**A**) TEM micrographs of cells obtained from control (0 mM of copper), treatment (0.00048× 0.5× and 1× MIC of copper) groups of *A. baumannii* strains AB003. Black arrows indicate pilus-like structures on the cell surface of *A. baumannii*. White arrows show physically damaged areas of a copper-treated cell. The scale bars represent 0.5 nm. (**B**,**C**) Cell couting was determined after 30 mins of treatments with difference concentrations of copper. ns; no statistical significant difference, *** *p*-values < 0.001 (statistical analysis by using one-way ANOVA).

The copper-tolerant strain AB003 that contained four copper-related clusters (Table S7) was selected as a representative to study the morphological changes and cell viability when treated with various concentrations of copper (Figure 2A). At 4.8 µM of copper, the morphology of *A. baumannii* cells remained undamaged even after 30 min of incubation at 37 ℃. Control and treatment at 4.8 µM still have the ability to produce appendages. In contrast to control, only a small number of pili could be found in the AB003 cells treated with 5 mM and 10 mM of copper (Figure 2A). The damaged cell envelopes of AB003 were detected after the treatment of a toxic concentration (10 mM) of copper. This physical damage was consistent with cell viability analysis that found no significant differences among cell survival (CFU/mL) in the control and the two treatment groups at 4.8 µM and 5 mM of copper (Figure 2B,C). While, the treatment at 10 mM of copper reduced the cell number by a 2-log reduction (*p* < 0.001) (Figure 2B,C).

### 3.5. Whole Genome Sequencing (WGS) and Computational Analysis of AB003

**Figure 3:**
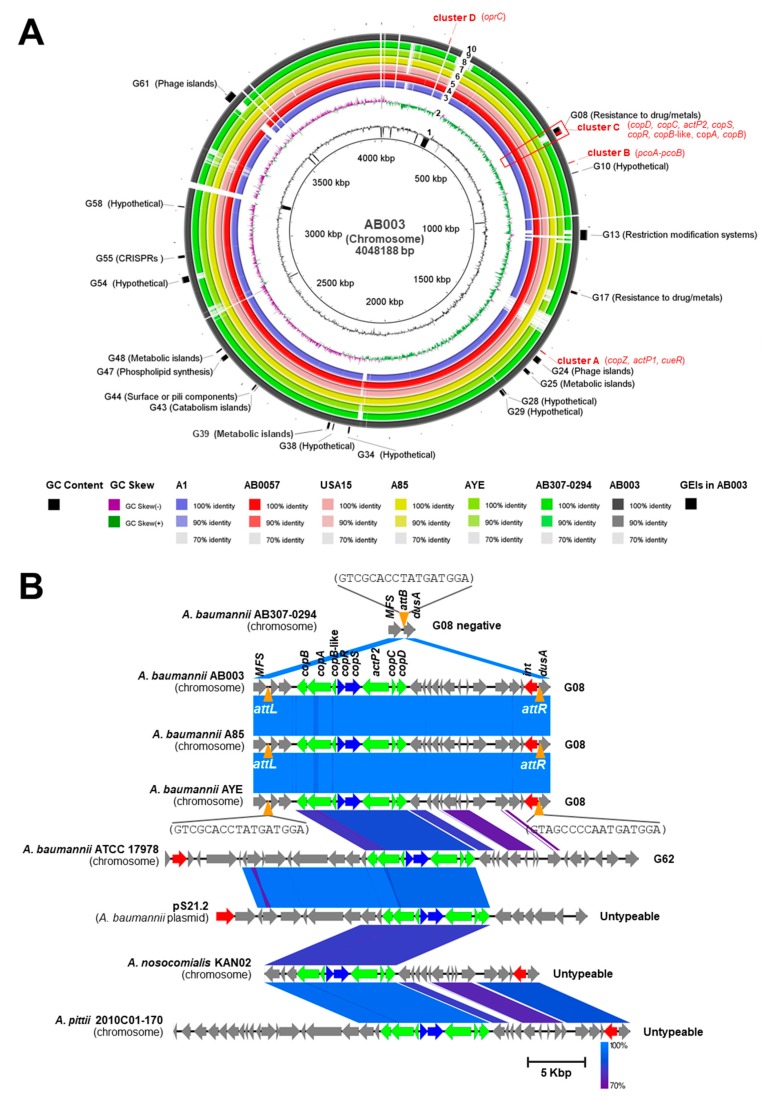
Whole genome analysis and comparision of genomic islands (GEIs) carrying copper-related genes identified in AB003 chromosome. (**A**) Graphical circular map of the chromosomal comparison of AB003 with other related strains, from inner to outer ring, rings 1 and 2 show GC (guanine-cytosine) content (black) and GC skew (purple/green) of AB003 chromosome. Rings 3 to 9 represent chromosomes of A1 (CP010781.1), AB0057 (CP001182.2), USA15 (CP020595.1), A85 (CP021782.1), AYE (CU459141.1), AB307-0294 (CP001172.2), and AB003, respectively. GEIs (black) and copper-related genes (red) located on AB003 chromosome are labelled in ring 10. The gaps in the circles illustrate regions of low or no similarity. The color intensity in each ring represents the BLASTn match identity. (**B**) Comparisons of GEIs harbored copper-related genes (G08, G62, and untypeable) extracted from both *Acinetobacter* spp. chromosome and plasmid. The orange inverted triangle represents the attachment site *attB* identified in *A. baumannii* AB307-0294. Orange triangles represent left end and right end attachment sites (*aatL* and *attR*) of G08. Strains and plasmid used for GEIs comparisions were AB307-0294 (CP001172, region: 3181958..3184295), A85 (CP021782, region: 666489..692480), AYE (CU459141, region: 3238701..3264693), ATCC 17978 (CP018664, region: 802734..844183), pS21.2 (MG954377, region: 65949..98419), KAN02 (CP036171, region: 3311588..3335626) and 2010C01-170 (CP029489, region: 3526129..3566104).

To provide insight into the genome characteristics involved in copper tolerance, a representative isolate AB003 was chosen for WGS analysis. After assembly, polishing and quality control, the assembled genome contained 57 contigs (GC content of 38.83%.), of which the largest contig consisted of 934,925 bp and the length of N50 was 223,399 bp. The ordered chromosome of AB003 was found to contain a 4,048,188 bp and identified 3735 coding sequences (CDSs), 5 rRNAs, and 65 tRNAs (see Supplementary Materials for the GenBank file, AB003.gbk). We identified 14 copper-related genes in AB003 and their predicted functions are showed in Table S2. Overall, the identified copper-related genes of AB003 encoded for copper-responsive transcriptional regulators (CueR, CopR, and CopS), copper efflux pumps (ActP1 and ActP2), copper chaperons (CopZ and CopC) and copper oxidizing enzymes (PcoA and CopA). We also divided putative copper-related genes identified in AB003 into four clusters (A-D) as described above (Table S2 and Figure 3A)

Cluster A contained three putative copper tolerance genes (*cueR*, *actP1*, and *copZ*), and cluster B carried two functional genes, *pcoA*, and *pcoB*. Cluster C that might be controlled by two-component regulators (CopRS) contained six structural genes (*copB*, *copA*, *copB*-like, *actP2*, *copC*, and *copD*). The fourth cluster (D) harbored gene encoding for TonB-dependent copper receptor/transporter, *oprC*. The comparisons of identified copper-related genes in AB003 and that in two *A. baumannii* reference strains revealed that these genes were highly conserved among analyzed *A. baumannii* strains (Table S2).

Genomic islands (GEIs) have been previously studied and 63 chromosomal loci (namely, G01-G63) have been identified in reference strains of *A. baumannii* such as AB0057, AYE, ACICU, and ATCC17978 [15]. We identified GEIs in chromosomal ordered contigs of AB003 and detected 19 GEIs (Figure 3A and Table S8). For instance, GEIs involved in resistance to metals (G08), restriction-modification systems (G13), metabolism (G25), surface or pili components (G44), phospholipid synthesis (G47), CRISPRs (G55), and phage island (G61) were identified on AB003 chromosome (Figure 3A and Table S8). Among four clusters of copper-related genes, we found that eight genes of cluster C were harbored on G08 (Figure 3A). GEIs are normally inserted at specific genomic sites and flanked by direct repeats that are important for their integration and excision. To detect these features, BLASTn comparison of G08, G62, and untypeable copper-carried GEIs were determined (Figure 3B). The G08 observed in AB003 was closely related to G08 identified in *A. baumannii* A85 and AYE (100% coverage and 99% identity) (Figure 3B). The comparison of G08 and G08-negative *A. baumannii* AB307-0294 is shown in Figure 3B. This revealed that the gene encoding for tRNA-dihydrouridine synthase A enzyme (*dusA*) carried the attachment or integration site (*attB*) that could be integrated by G08. We also found that G08 of AB003 carried the integrase gene (*int*) and was flanked by the 17-bp attachment sequences at the left (*attL*) and right (*attR*) ends. These two 17-bp attachment sequences were conserved among examined strains (Figure 3B).

Interestingly, database search revealed that cluster C-carried untypeable GEIs were found across different *Acinetobacter* spp. such as *A. nosocomialis* (CP036171.1), *A. schindleri* (CP019041.1), *A. soli* (CP016896.1), and *A. junii* (CP019041.1). Moreover, these GEIs were normally found to be located on chromosomes, except that in some strains of *A. lwoffii* (pALWED3.1; KX528687.1), *A. schindleri* (p1AsACE; CP015616.1) and *A. baumannii* (pS21.2; MG954377) were found to be carried on plasmids.

### 3.6. Expression of Copper-Related Genes in Response to Copper of AB003

**Figure 4:**
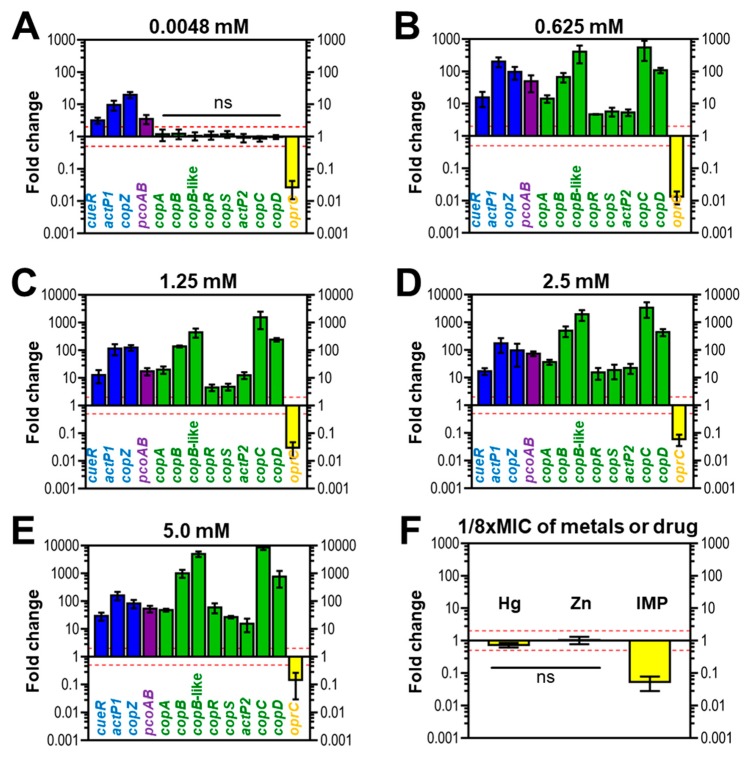
Gene expression profiles of putative copper-related genes of the representative strain AB003 in response to various copper levels. (**A**–**E**) mRNA expression fold-change of copper-related genes detected in genome of AB003 under (**A**) the micromolar (0.0048 mM or 4.8 µM) and (**B**–**E**) millimolar (0.625 to 5 mM) levels of copper treatments. (**F**) Fold-change expression of AB003 *oprC* in the presence of sub-MIC (1/8×) of Hg; mercury, Zn; zinc, and IMP; imipenem. Comparisions of relative fold-changes were analyzed by Student’s t test using a reference value of 1 (a housekeeping gene, *gyrB*). Relative fold-changes of all selected genes were significantly different (*p* < 0.05) when compared to controls except genes denoted as ns (ns: not statistical difference). Error bars show standard diviations of the mean (indipendence biological triplicates). The red dashed lines represent significant two-fold up (2×-relative fold-change) and down regulation (0.5×- relative fold-change) of each treatment condition.

Overall, copper treatment at a micromolar concentration (4.8 µM) affected the expression of genes located in three clusters (Figure 4A). In this condition, all genes in cluster A and B were up-regulated (≥2×-folds), while the *oprC* of cluster D (≤0.5×-folds) was down-regulated. Conversely, eight genes embedded in cluster C did not increase or decrease in response to copper addition at a micromolar level (0.5× < relative fold changes < 2×) (Figure 4A). Unlike treatment with micromolar copper concentration, all putative copper-related genes derived from four clusters of AB003 were responsive to copper exposure at millimolar levels of copper (0.625, 1.25, 2.5, and 5 mM) ≤ 0.5× or ≥ 2×-fold changes) (Figure 4B–E).

In cluster A, a transcriptional regulator gene, *cueR* was up-regulated ranging from 2- to 29-folds as illustrated in Figure 4A–E. While genes encoded for copper efflux pump and chaperone (*actP1* and *copZ*) were responsive by more than five-folds at a micromolar copper concentration (Figure 4A) and 100-folds after treatment with millimolar concentrations of copper (Figure 4B–E). Although a slight increase in *pcoAB* expression level was observed in cells challenged with 4.8 µM copper, then the reaching peak expression was found to be around 50-folds in treatments with millimolar concentrations of copper (Figure 4B–E). For cluster C, all genes were unresponsive to a supplementation with 4.8 µM copper (Figure 4A), while the exposures to millimolar copper concentrations resulted in upregulation of all genes. For example, *copC* mRNA showed an initial upregulation of ~ 500-fold in cells treated with 0.625 mM copper (Figure 4B), followed by a statistically significant change to approximately 1500-fold by exposure to 1.25 mM of copper (Figure 4C), and reaching the peak at around 8000-fold after treatment with 5 mM copper (Figure 4E). For cluster D, transcripts from *oprC* were decreased by 3- and 100-fold (relative fold changes; 0.3× and 0.01×) in challenge conditions of 4.8 µM and 0.625 mM copper, respectively (Figure 4A,B).

We also determined the transcriptional profiles of *oprC* in response to a sub-MIC of other metals like mercury, zinc as well as the antibiotic imipenem. Similar to the presence of copper (1/8× MIC) (Figure 4C), imipenem (1/8× MIC) (Figure 4F) suppressed the expression of *oprC* by 20-fold (0.05×-relative fold change). However, 1/8× MIC of mercury and zinc did not result in change in *oprC* expression (Figure 4F).

## 4. Discussions and Conclusions

The wide application of copper and copper-containing compounds in animal feed supplements, fungicides, and medical equipment can enhance the risk of copper contamination in the environment and is the factor that drives the selection and spread of copper-tolerant bacteria [1]. In the present work, copper-tolerant *A. baumannii* could be detected in both clinical and natural environmental isolates. Previous studies reported that copper tolerance was found in pathogenic bacteria isolated from environmental, food, and clinical samples [11,31,32]. We found a narrow range of copper MIC values (5 and 10 mM) for 407 *A. baumannii* isolates, by using a rich growth medium (MHA). This result is consistent with a current finding reported by Yang et al. [31], who showed that copper MICs of *Klebsiella pneumoniae*, *Citrobacter freundii*, and *Escherichia coli* were found in the range of 5–10 mM. Williams and co-workers determined the copper tolerance of *A. baumannii* in the minimal liquid medium and found that at micromolar levels copper can inhibit the growth of *A. baumannii* [11]. However, the susceptibility testing in minimal medium was not suitable because of the precipitation of copper at higher concentration [11].

Our analysis showed *cueR*, *pcoAB*, and *oprC* (representative genes of clusters A, B, and D, respectively) were harbored by all tested *A. baumannii* isolates, while *copRS* (cluster C) was detected in around 20% of all isolates. We hypothesized that the genes from these three clusters provide intrinsic copper tolerance (*cueR* and *pcoAB*) and utilization mechanisms (*oprC*), while *copRS* might be acquired by *A. baumannii*. This hypothesis was confirmed by our in silico analysis, which found that all complete genomes of *A. baumannii* were positive for *cueR*, *pcoAB*, and *oprC*, while *copRS* was found to be carried in only some strains. Moreover, the comparison between copper-tolerant phenotype and genotype revealed that the presence of acquired *copRS* (cluster C) was associated with the ability to tolerate copper. However, some copper-tolerant isolates (12 isolates) could not be detected *copRS*, suggesting that these isolates likely encode other copper tolerance mechanisms such as the production of extracellular substances that protect themselves from copper toxicity [33].

In this work, we studied the association between phenotypic tolerance to copper and antibiotics and found a slight relationship. There are low chances of finding the association between resistance phenotype to antibiotics and metals because one phenotype can be controlled by many genes. However, we found that the copper-tolerant phenotype was associated with resistance to amikacin. This finding could be explained by the possibility of genes involved in the resistance to both compounds being co-located on the same chromosomal region or plasmids. For example, we searched the database of *A. baumannii* plasmids and found that plasmid pC13-2 (KU549175.1) harbored both amikacin-resistant and copper-related genes. Moreover, in the present work, we found the association between genes confers tolerance to copper (*copRS*) and antibiotics (*bla*_NDM-1_). Likewise, the published work of Yang et al. [31] reported that the copper-related gene can be co-transferred with the cabarpenemase gene on the same conjugative plasmids among *Enterobacteriaceae*. Together, these findings indicated that the co-spreading of tolerance to heavy metals and antibiotics can be found in clinical strains of pathogenic bacteria.

The mechanisms by which copper can kill and destroy bacterial cells have been reported to be involved in the direct inactivation of cellular components or induction of ROS productions [3]. In this work, the observation of external morphology revealed that pilus-like surface appendages were inhibited by exposure to the sub-MIC of copper (5 mM), while the MIC level of copper (10 mM) caused morphological changes of cell envelope. We proposed that these external morphological changes might be the results of the induction of copper-derived ROSs or the interaction of copper with cellular compartments which caused further cell damage. However, copper toxicity depends on the type of copper involved, such as metallic copper surfaces and copper solutions [3,34]. For example, Espírito Santo et al. reported that bacteria are rapidly killed on metallic copper surfaces that resulted from extensive membrane damage and loss of cell integrity [34].

As reported by previous studies, putative copper-related genes were located on chromosomal regions of *A. baumannii* [11,12,13]. To identify and localize copper-related genes, we sequenced the genome of *A. baumannii* AB003 and found fourteen copper-related genes located in four chromosomal regions (clusters A, B, C, and D). Moreover, our computational analysis revealed that cluster C of AB003 contained eight copper-related genes, which are embedded in genomic island (G08). This finding is consistent with other that have reported *A. baumannii* strain AYE and ATCC17978-harbored copper tolerance genes on G08 and G62, respectively [13,15]. Similar to other prophages and genomic islands that have been reported in *A. baumannii* [35], G08 detected in AB003 was integrated within the tRNA-dihydrouridine synthase A (dusA) gene. Here, our bioinformatics analysis also found that G08 and untypeable GEIs carried cluster C were detected on conjugative plasmids of various *Acinetobacter* spp. Likewise, copper resistance operons have been described in other pathogenic bacteria and these operons can be acquired by bacteria [3,31,36]. In this context, our data suggested that this mobile genetic element was widely disseminated in the genus *Acinetobacter* and might be acquired through horizontal gene transfer.

Overall, we found that the exposure to various concentrations of copper resulted in a significant induction of genes located in all four clusters. It is interesting to note that intrinsic copper-related genes (clusters A, B, and D) could be induced by copper addition at the micromolar concentration (4.8 µM) and millimolar concentrations (0.625 to 5 mM) while, the acquired copper-related cluster C required the supplementation of millimolar concentrations. In agreement with a previous study, genes expressions of cluster C (*copB*-like, *copRS*, *actP2* of ABUW_3325) occurred at higher concentrations of copper than required for induction of cluster A (*actP1*; ABUW_2707) [11].

**Figure 5:**
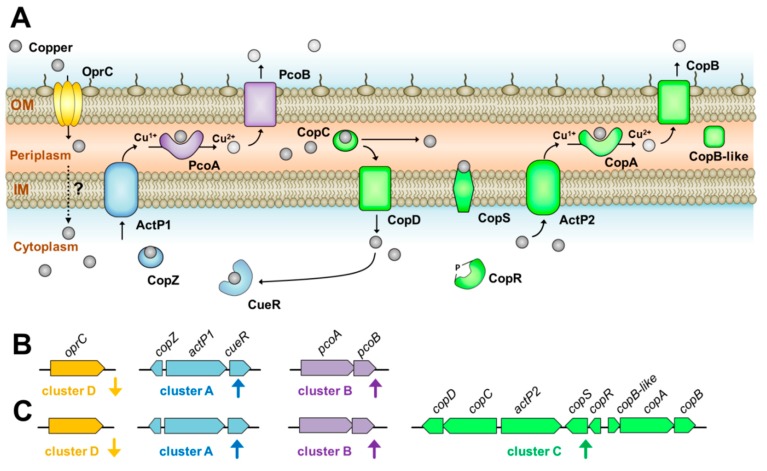
Schematic representation of various genes identified in *A. baumannii* AB003 and their proposed role in copper tolerance based on genome, transcriptional, and bioinformatic analysis performed in this work. (**A**) The inner membrane (IM) and outer membrane (OM) are demonstrated as phospholipid bilayers, copper are depicted in grey ovals and directions of copper transport are indicated by arrows. In silico analysis was performed in order to predict the functions of each determinant, while subcellular localizations and transmembrane domains of copper-related proteins were determined according to the methodology described above. (**B**,**C**) The genetic architecture of four copper-responsive clusters are shown. (**B**) In the presence of a micromolar concentration of copper, proposed intrinsic copper-related genes (clusters A, B, and D) were regulated. (**C**) While in millimolar concentrations of copper, additional (cluster C) together with intrinsic copper-related genes were activated to promote the enzymatic detoxification and active efflux of copper.

In *Pseudomonas aeruginosa*, OprC is an outer membrane porin, involved in utilization of copper [24]. Interestingly, expression of the *oprC* homologue identified in AB003 was suppressed in the presence of copper, suggesting that this regulation was used as the first line of defense to prevent the influx of toxic copper by *A. baumannii* AB003 (Figure 5). In *E. coli*, when the intracellular copper concentration is sufficient, bacteria normally encode the copper sensor (CueR), P-type ATPase efflux pump (CopA) and multi-copper oxidizing enzyme (CueO) to recognize copper, transfer copper from cytoplasm to periplasm and detoxify copper, respectively [2,3]. Similarly, in cluster A of AB003, genes homologous to *cueR* and efflux pump were identified, while a CueO homologue could not be detected. Although cluster A of AB003 lacked a copper oxidase homologue, it appeared that cluster B encoded this homologue (PcoA) together with a copper binding outer membrane porin (PcoB). Based on this finding, we hypothesized that copper tolerance proteins encoded from cluster A and B worked together in detoxifying copper and extruding detoxified periplasmic copper out of the cell (Figure 5). Additional copper-resistance in the periplasm of *E. coli* and *Pseudomonas* spp. is mediated by *pco* or *cop* genes [3], which were homologous to the genes in cluster C detected on the AB003 genome. Unlike Pco and Cop systems, AB003 cluster C encoded both the second P-type ATPase (ActP2) and CopAB homologs that might play a dual copper tolerance in both cytoplasm and periplasm (Figure 5). However, our transcriptional analysis showed that *copC* and *copD* genes believed to be responsible for importing copper into the cytoplasm [3], were gradually over-expressed when treated with high concentrations of copper. We hypothesized that copper imported by the CopCD might be trapped by the copper-dependent cytoplasmic regulator CueR, resulting in expression of *copZ* and *actP1* genes encoding a cytoplasmic copper efflux system (Figure 5).

Cross-resistance occurs when a single system or resistance gene confers resistance to both antibiotics and metals [37,38]. Here, we demonstrated that the *oprC*, a putative gene encoded for outer membrane porin involved in copper uptake, were responsive to supplementations of copper as well as imipenem, suggesting that this gene implicated in conferring cross-resistance to both metal and antibiotic in *A. baumannii*. In addition to cross-resistance, copper resistance proteins, such as copper-responsive P-type ATPases efflux pump (ActP1) and outer membrane porin (OprC), have been elucidated in the pathogenesis of *A. baumannii* [14,39]. Our study found that *actP1* and *oprC* were detected in all analyzed strains, suggesting that these copper-related genes were generally encoded by *A. baumannii* to act as cross-resistance determinants and virulence factors.

In conclusion, our results revealed that the prevalence of copper-tolerant *A. baumannii* remains low; however, we could found copper tolerance in both clinical and environmental isolates. Moreover, *A. baumannii* harbored copper-related genes encoded from proposed intrinsic clusters (A, B, and D) and proposed additional cluster C which associated with copper-tolerant phenotype and the presence of antibiotic resistance genes. The genome and gene expression analysis of *A. baumannii* AB003 showed that intrinsic copper tolerance mechanisms appeared to be activated in the additions of copper, while an acquired tolerance (cluster C) was induced in the presence of high concentrations of copper. As copper tolerance mechanisms are responsible for resistance to antibiotic and metal as well as important factors for full bacterial virulence during infection. Thus, the presence of many copper-tolerant determinants in *A. baumannii* could have a concern for copper application in hospitals and environments, and for effectively controlling bacteria through specific targeting of copper-resistant systems.

## Figures and Tables

**Table 1:** The copper susceptibility of *A. baumannii* clinical and environmental isolates carrying difference patterns of copper-related genes.

Patterns of Copper-Related Genes ^a^	Total Isolates (%)	Copper Susceptible Isolates (MIC = 5 mM) (%)	Copper-Tolerant Isolates ^b^ (MIC = 10 mM) (%)
All isolates	n = 407 (100)	311 (100)	96 (100)
*cueR*^+^/*pcoAB*^+^*/copRS*^-^*/oprC*^+^	323 (79.4)	311 (100)	12 (12.5)
*cueR*^+^/*pcoAB*^+^*/copRS*^+^*/oprC*^+^	84 (20.6)	0 (0)	84 (87.5) *
Environmental isolates	n = 13 (100)	7 (100)	6 (100)
*cueR*^+^/*pcoAB*^+^*/copRS*^-^*/oprC*^+^	7 (53.8)	7 (100)	0 (0)
*cueR*^+^/*pcoAB*^+^*/copRS*^+^*/oprC*^+^	6 (46.2)	0 (0)	6 (100) *
Clinical isolates	n = 394 (100)	304 (100)	90 (100)
*cueR*^+^/*pcoAB*^+^*/copRS*^-^*/oprC*^+^	316 (80.2)	304 (100)	12 (13.3)
*cueR*^+^/*pcoAB*^+^*/copRS*^+^*/oprC*^+^	78 (19.8)	0 (0)	78 (86.7) *

^a^ The studied genes *cueR*, *pcoAB*, *copRS*, and *oprC* were chosen as representatives of genes located on cluster A, B, C and D, respectively. ^b^ Relationships between copper-tolerant phenotype and patterns of copper-related genes were statistically determined by using Fisher’s exact test. * Asterisk represents statistical associations of the present of *copRS* and copper-tolerant phenotype (*p* < 0.001).

## Supplementary Materials

Supplementary materials can be found at https://www.mdpi.com/2076-0817/9/1/60/s1. Table S1. Characteristics of 407 *Acinetobacter baumannii* isolated from environmental and clinical samples. Table S2. Putative chromosomal genes involved in copper tolerance and utilization mechanisms identified in the *Acinetobacter baumannii* reference strains and the representative strain AB003. Table S3. Primer for detection of copper-related genes located on four clusters. Table S4. List of primer and conditions used for RT-qPCR analysis in this study. Table S5. In silico detection of selected representative genes located on cluster A-D in 153 complete genome sequenced *A. baumannii*. Table S6. In silico detection of selected representative genes located on cluster A-D in complete sequencing of *A. baumannii* plasmids obtained from the NCBI database. Table S7. Characteristics of the representative *Acinetobacter baumannii* strain AB003. AB003.gbk. Table S8. Lists of the genomic islands (GEIs) detected on AB003 chromosome and pairwise sequence alignment with reference strain AYE.
